# The association between the *miR-146a* rs2910164 C>G polymorphism and Kawasaki disease in a southern Chinese population

**DOI:** 10.1042/BSR20180749

**Published:** 2018-07-03

**Authors:** Li Zhang, Jinxin Wang, Di Che, Yanfei Wang, Xing Rong, Lei Pi, Yufen Xu, Wei Li, Ping Huang, Maoping Chu, Xiaoqiong Gu

**Affiliations:** 1 Department of Cardiology, Guangzhou Women and Children’s Medical Center, Guangzhou Medical University, Guangzhou 510623, Guangdong, China; 2 Children’s Heart Center, The Second Affiliated Hospital and Yuying Children’s Hospital, Institute of Cardiovascular Development and Translational Medicine, Wenzhou Medical University, Wenzhou 325027, Zhejiang, China; 3 Department of Clinical Lab and Biological Resource Bank, Guangzhou Institute of Pediatrics, Guangzhou Women and Children’s Medical Center, Guangzhou Medical University, Guangzhou 510623, Guangdong, China

**Keywords:** Kawasaki disease, miRNA-146a, single nucleotide polymorphism, susceptibility

## Abstract

*miR-146a* plays a critical role in innate immune and inflammatory responses. Kawasaki disease involves immune-mediated inflammatory responses, which leads to vascular endothelial injury. However, there has been no study on the association between the *miR-146a* rs2910164 C>G polymorphism and Kawasaki disease risk. We enrolled 532 Kawasaki disease patients and 623 healthy controls from southern Chinese population, and the *miR-146a* rs2910164 C>G polymorphism was genotyped by the TaqMan method. There was no evidence that this polymorphism was associated with Kawasaki disease. Stratified analysis also showed no significant association. The present study indicates that the *miR-146a* rs2910164 C>G polymorphism may not be associated with Kawasaki disease in the southern Chinese population. Larger multicenter studies are needed to confirm our conclusions.

## Introduction

Kawasaki disease, a systemic syndrome, involves vasculitis associated with fever and primarily endangers young children (age less than 5 years) [1]. This disease has become the leading cause of acquired heart disease in children in developed countries [2]. The incidences are highest amongst the Asians and Pacific Islanders [3]. Epidemiological studies have shown a strong tendency to genetic predisposition in Kawasaki disease. The etiology of Kawasaki disease is not entirely clear. This disease is now known to be an immune-mediated inflammatory response, which leads to vascular endothelial injury [4]. Coronary lesions are the main complication of Kawasaki disease. Moreover, as the proportion of typical Kawasaki disease decreased and incomplete Kawasaki disease increased [5], the difficulty of diagnosis also increased.

miRNAs are 20–25 nts in length and are non-coding ssRNA molecules that regulate gene expression via mRNA. miRNAs play a role in tumorigenesis [6], diabetes [7], cardiac hypertrophy [8], and ischemic heart failure [9]. Previously, many miRNAs have been used as biomarkers for a variety of diseases, and some of these molecules have been identified as diagnostic biomarkers for Kawasaki disease, such as *miR-200c* and *miR-371-5p* [10]. We noted that *miR-146a* plays a pivotal role in innate immune [11,12] and inflammatory responses [13–15]. Innate immune and inflammatory responses are the basic pathogenesis of Kawasaki disease [16]. These findings prompted us to examine whether the *miR-146a* rs2910164 C>G polymorphism is associated with Kawasaki disease and whether the rs2910164 C>G polymorphism can be used as a potential risk factor or as a biomedical indicator. However, there was no previous research on the association between the *miR-146a* rs2910164 C>G polymorphism and Kawasaki disease; thus, we carried out the present study.

## Materials and methods

### Study subjects

The present study included 532 children who were diagnosed with Kawasaki disease from the Guangzhou Women and Children’s Medical Center from January 2012 to December 2017 [17]. Diagnostic criteria referred to the 2004 American Heart Association recommendations [2], and 623 age- and gender-matched healthy children at the hospital in the same period for physical examination were also analyzed. Blood samples were provided by the Clinical Biological Resource Bank of the Guangzhou Women’s and Children’s Medical Center. Information on each case, such as age, gender, coronary artery lesion (CAL), and coronary artery aneurysm (CAA), were collected. CAL and CAA were also graded according to the 2004 American Heart Association recommendations. The present study obtained the consent of the participants’ parents or guardians and was approved by the Guangzhou Women and Children’s Medical Center Medical Ethics Committee (ethics number: 2014073009).

### Genotyping

We extracted the DNA from the blood samples. The extracted DNA was placed in a −80°C refrigerator until use. For genotyping of the rs2910164 C>G polymorphism, we used ABI Q6 instrument (Applied Biosystems) and TaqMan assays [18–20]. The genotypes of cases and controls were determined during this process. For quality control, each 384-well plate contained eight samples without DNA but with the same amount of distilled water.

### Statistical analysis

The differences in the distribution of data of children with Kawasaki disease and healthy controls were assessed by the *χ^2^* test. Odds ratios (ORs) and 95% confidence intervals (CIs) of homozygotes (GG compared with CC), heterozygotes (CG compared with CC), a recessive model (GG compared with CC + CG), and a dominant model (CC + CG compared with CC) were used to assess the association between the *miR-146a* rs2910164 C>G polymorphism and Kawasaki disease. Univariate and multivariate regression models were used to calculate the OR values. Additionally, the association between the rs2910164 C>G polymorphism and Kawasaki disease was stratified by gender, age, CAL, and CAA. The statistics were performed by SAS software (version 9.4, SAS Institute, Cary, NC, U.S.A.).

## Results

### Demographic characteristics

The demographic characteristics of the study subjects are shown in Supplementary Table S1. The average age of onset of the cases was 28.39 ± 24.68 months, 68.61% were male, the male to female ratio was 2.19:1, CAL accounted for 31.58%, and CAA accounted for 9.59%. The average age of the controls was 28.48 ± 25.33 months, and 64.53% were male (*P*=0.143). There were no significant differences in age and gender between Kawasaki disease patients and controls (*P*=0.602). Gender, age, and coronary artery outcomes were adjusted for further analysis.

### Genotype distributions

The rs2910164 allele frequencies in the case group and control group are shown in Table 1, as well as the association between the rs2910164 C>G polymorphism and Kawasaki disease risk. The data showed that rs2910164 C>G was not significantly associated with Kawasaki disease compared with the controls (CG compared with CC: OR = 1.01, 95% CI = 0.79–1.30; GG compared with CC: OR = 0.95, 95% CI = 0.66–1.36; GG compared with CC + CG: OR = 0.94, 95% CI = 0.68–1.31, CC + CG compared with CC: OR = 1.00, 95% CI = 0.78–1.27).

**Table 1:** Genotype distributions of the *miR-146a* rs2910164 C>G polymorphism and Kawasaki disease susceptibility

Genotype	Cases (*n*=532)	Controls (*n*=616)	P*	Crude OR (95% CI)	*P*	Adjusted OR (95% CI)†	*P*†
rs2910164 (HWE = 0.902)							
CC	199 (37.41)	230 (37.34)		1.00		1.00	
CG	258 (48.50)	294 (47.73)		1.03 (0.80–1.33)	0.807	1.01 (0.79–1.30)	0.924
GG	75 (14.10)	92 (14.94)		0.96 (0.67–1.37)	0.818	0.95 (0.66–1.36)	0.763
Additive			0.917	0.98 (0.83–1.16)	0.823	0.98 (0.83–1.16)	0.834
Dominant	333 (62.59)	386 (62.66)	0.981	1.00 (0.79–1.27)	0.981	1.00 (0.78–1.27)	0.979
Recessive	457 (85.90)	524 (85.06)	0.688	0.94 (0.67–1.30)	0.689	0.94 (0.68–1.31)	0.712

* *χ^2^* test for genotype distributions between Kawasaki disease patients and controls.

† Adjusted for age and gender.

HWE: Hardy-Weinberg equilibrium.

### Stratification analysis

The stratification analyses were based on gender, age, and coronary artery abnormality. It was indicated that the rs2910164 C>G polymorphism had no significant association with Kawasaki disease risk in different age groups, between males and females, CAA and NCAA, CAL and NCAL (Table 2).

**Table 2:** Stratification analysis for the association between the *miR-146a* rs2910164 C>G polymorphism and Kawasaki disease susceptibility

Variables	CC	CG/GG	Crude OR	*P*	Adjusted OR*	P*
	Cases/controls		(95% CI)		(95% CI)	
Age, months						
<12	46/66	91/98	1.33 (0.83–2.14)	0.234	1.32 (0.82–2.13)	0.254
12–60	133/144	218/247	0.96 (0.71–1.29)	0.765	0.96 (0.71–1.29)	0.765
>60	20/20	24/41	0.59 (0.26–1.30)	0.189	0.55 (0.24–1.25)	0.154
Gender						
Females	62/84	105/137	1.04 (0.69–1.57)	0.859	0.99 (0.65–1.52)	0.978
Males	137/146	228/249	0.98 (0.73–1.31)	0.871	0.97 (0.72–1.31)	0.856
CAA						
CAA	17/230	34/386	1.19 (0.65–2.18)	0.570	1.18 (0.65–2.17)	0.585
NCAA	182/230	299/386	0.98 (0.77–1.25)	0.865	0.98 (0.77–1.25)	0.873
CAL						
CAL	57/230	111/386	1.16 (0.81–1.66)	0.416	1.16 (0.81–1.67)	0.414
NCAL	142/230	222/386	0.93 (0.71–1.22)	0.601	0.93 (0.71–1.22)	0.597

* Adjusted for age and gender.

## Discussion

Genetic susceptibility has become a concern for Kawasaki disease research, but few studies have examined the association of miRNA polymorphisms with Kawasaki disease. He et al. [21] reported that *miR-483* contributes to coronary artery abnormalities in Kawasaki disease; Shimizu et al. [22] reported that *miR-145* affects patients with Kawasaki by the TGF-β pathway; and Yun et al. [10] reported that *miR-200c* and *miR-371-5p* were involved in Kawasaki disease by regulating the inflammatory response.

By the means of promoter analysis, *miR-146a* was found to be an NF-κB-dependent gene and base pairs with sequences in the 3′-UTRs of the *TNF receptor-associated factor 6* and *IL-1 receptor-associated kinase 1* genes [12,13], which played an important role in Kawasaki disease-induced vasculitis [23,24]. *miR-146a* expression provided a novel mechanism for the negative regulation of severe inflammation [12,13,25], and *miR-146a* was involved in vascular endothelial cell senescence through NADPH oxidase-4 (NOX4) [26]. Thus, we wondered whether there was an association between *miR-146a* and Kawasaki disease. Furthermore, *miR-146a* rs2910164 C>G was found to increase the HBV-related hepatocellular carcinoma risk especially in the Chinese population, and meta-analysis results suggested that the rs2910164 C>G polymorphism was associated with increased breast cancer risk [27,28]. Therefore, we carried out the present study with a relatively large sample size, including 532 cases and 623 healthy children from southern Chinese population, and *miR-146a* was selected as the study subject.

The present study showed that *miR-146a* rs2910164 C>G was not associated with Kawasaki disease and was not related to other parameters such as gender, age, CAL, and CAA. Kawasaki disease is more prevalent in males and young children, less than 5 years old. The results of this experiment showed that the rates of the *miR-146a* rs2910164 C>G polymorphism in different gender groups and age groups had no difference. The major complications in Kawasaki disease are CALs and CAAs. In stratified analysis, there were also no significant correlations between the *miR-146a* rs2910164 C>G polymorphism and CAL or CAA risk in Kawasaki disease. Our current study showed that the *miR-146a* rs2910164 C>G polymorphism may not be suitable as a biomarker for the diagnosis or prognosis of Kawasaki disease.

The current study has some limitations. First, the analysis of Kawasaki disease included gender, age, CAL, and CAA, but other factors such as family history and birth history [5], were not taken into account in the stratification analysis due to the lack of information. These factors may affect the results if the information was available. Second, the study was limited to southern Chinese population, and cases and controls from other ethnic groups were not assessed. Given the vast differences in the incidence of Kawasaki disease amongst different races, the distribution and the function of *miR-146a* in various populations require further study.

In conclusion, we carried out a case–control study with a relatively large sample size with 532 cases and 623 healthy controls from southern Chinese population. The association between the *miR-146a* rs2910164 C>G polymorphism and Kawasaki disease, as well as other parameters such as coronary artery complications, were not significant. Larger multicenter studies are needed.

## Supporting information

**Supplemental Table 1:** Frequency distribution of selected variables for cases with Kawasaki disease and controls

## Abbreviations

CAA: coronary artery aneurysm
CAL: coronary artery lesion
CI: confidence interval
HBV: hepatitis B virus
IL-1: interleukin-1
NCAA: non-coronary artery aneurysm
NCAL: non-coronary artery lesion
OR: odds ratio
TGF-β: transforming growth factor-β
TNF: tumor necrosis factor
