# Supplementary material for: The association between the miR-146a rs2910164 C>G polymorphism and Kawasaki disease in a southern Chinese population
Source: Biosci Rep. 2018 Jul 3;38(4):BSR20180749. doi: 10.1042/BSR20180749 (PMC6028755; doi:10.1042/BSR20180749)
Supplement: Supplementary file 1 [file bsr20180749_Supp1.pdf]

Supplemental Table 1. Frequency distribution of selected variables for cases with Kawasaki disease and controls

Variables	Cases (n=532)		Controls (n=623)		P [a]
	No.	%	No.	%	
Age range, month	1.00-166.0		0.07-166		0.602
Mean ± SD	28.39 ± 24.68		28.48 ± 25.33		
<12	137	25.75	165	26.48	
12-60	351	65.98	397	63.72	
>60	44	8.27	61	9.79	
Gender					0.143
Female	167	31.39	221	35.47	
Male	365	68.61	402	64.53	
Coronary artery outcomes1					
CAA	51	9.59			
NCAA	481	90.41			
Coronary artery outcomes2					
CAL	168	31.58			
NCAL	364	68.42			

CAA, coronary artery aneurysm; CAL, coronary artery lesion.

[a] Two-sided χ^2 test for distributions between cases and controls.
